# A Rare and Life-Threatening Complication of Salmonella Typhi Infection: A Case Report From India Highlighting Diagnostic and Therapeutic Challenges in Salmonella Sepsis

**DOI:** 10.7759/cureus.76234

**Published:** 2024-12-23

**Authors:** Jaziya Jabeen, M. Ardra, Chithra Valsan, John Paul, Cherish Paul

**Affiliations:** 1 Cardiology, Royal Cornwall Hospital, Cornwall, GBR; 2 Microbiology, Jubilee Mission Medical College & Research Institute, Thrissur, IND; 3 Critical Care, Jubilee Mission Medical College & Research Institute, Thrissur, IND

**Keywords:** diagnostics, dic, salmonella typhi, sepsis, typhoid fever

## Abstract

Salmonella infections are widely known to cause gastroenteritis, especially in areas of poor hygiene and sanitation. Common symptoms include sustained fever, chills, and abdominal pain. Sepsis, disseminated intravascular coagulation (DIC), various neurological manifestations, and multiorgan failure are other uncommon presentations. Raising appropriate awareness about its clinical spectrum is therefore crucial, even in the absence of typical symptoms.

The following case of a 34-year-old Indian female who developed *Salmonella Typhi*-induced sepsis addresses various challenges involved in diagnosing and treating the condition. The case was successfully managed with rapid diagnostics, targeted antibiotic therapy, and supportive care. Recognizing the condition early and providing necessary treatment is therefore vital to preventing substantial morbidity and mortality. To improve outcomes for critically ill patients, it is important that the causative organism be identified quickly and appropriate treatment be commenced. Here, we present a case of Salmonella sepsis complicated by DIC, its clinical course, and the diagnostic and treatment modalities we have followed.

## Introduction

Salmonella infections are widely known to cause gastroenteritis, especially in areas of poor hygiene and sanitation. Typhoid fever is a life-threatening acute febrile illness caused by *Salmonella Typhi*. The majority of typhoid fever cases are reported in South Asia, Southeast Asia, and Sub-Saharan Africa, owing to poor sanitation and a lack of clean water [1]. The disease affects around 11-21 million people every year, causing approximately 128,000-161,000 deaths worldwide [2].

Clinical presentations of typhoid fever are widely variable. The majority of the cases present as febrile illness, accompanied by gastrointestinal symptoms, and may proceed to severe complications including intestinal perforation, meningitis, and multi-organ dysfunction, which is reported with increased frequency among high-risk groups including children, immunocompromised patients, and those exposed to multidrug-resistant and extensively drug-resistant (MDR or XDR) strains of *S. Typhi *[3, 4].

The non-specific symptom profile of *Salmonella Typhi *infections, with symptoms that overlap with other tropical diseases such as malaria, dengue fever, and leptospirosis, also complicates clinical diagnosis in endemic areas. In endemic regions, the diagnosis of typhoid fever can be challenging due to its nonspecific presentation [5-7]. Sepsis is a severe manifestation of typhoid fever, which results from a dysregulated host response to systemic infection and remains a leading cause of mortality among hospitalized patients. Without treatment, the case fatality rate of typhoid fever is 10-30%, which with appropriate therapy is dropping to 1-4% [7]. Rapid and precise diagnosis is crucial in these settings, where even short delays in treatment significantly increase the possibility of severe outcomes, including multiple organ dysfunction syndrome (MODS) and disseminated intravascular coagulation (DIC) [8].

According to the survival sepsis guidelines, initiating the correct antibiotic within the first hour of hospitalization with rapid identification of the causative organisms significantly improves the chances of a positive outcome [9]. In severe cases, ceftriaxone is still the primary treatment cornerstone, but the development of resistance patterns has driven calls for antibiotic stewardship as well as innovation in newer therapies [10].

Molecular diagnostics continue to improve with the aid of multiplex polymerase chain reaction (PCR) and panels for the identification of cultures from blood; this helps in achieving early detection of *Salmonella Typhi *as well as appropriate treatment targeting [11].

Blood culture remains the mainstay for definitive antibiotic therapy, although the positivity is between 40%-80% and sensitivity depends on various factors, including the timing of the initial culture, volume of the blood sample, site of culture, transport, and prior antimicrobial use [3, 12, 13]. 

The clinical challenges and therapeutic strategies for managing *S. Typhi*-induced sepsis complicated by DIC and MODS are illustrated in this case report. It underscores the critical role of multidisciplinary care, rapid diagnostics, and aggressive management to salvage life, particularly in resource-constrained settings.

## Case presentation

A 34-year-old Indian female was brought to the emergency department (ED) with a history of high-grade fever for five days and an episode of loose stool. She experienced two episodes of seizures at home on the day of admission and was brought to the hospital in a state of post-ictal drowsiness. Further, the patient also had one episode of seizure at the ED. The patient had a history of ductal carcinoma breast in 2018, for which mastectomy was performed, and has been on tamoxifen since then.

The patient was shifted from the ED to the Multidisciplinary Critical Care Unit (MCCU) of the hospital for further management. The patient developed ecchymotic patches on her right arm and left forearm on the same day. On Day 1 (D1) of admission, along with routine bloods, the following investigations were sent to the department of microbiology: real-time PCR for Leptospira and Dengue (Truenat, Molbio Diagnostics, Verna, India) and ELISA for Dengue NS1 antigen, Dengue IgM antibody, and Leptospira IgM antibody, and results were negative. In view of sepsis with MODS, blood culture (automated BacT/Alert blood culture system, bioMérieux SA, Marcy-l'Étoile, France) and BioFire blood culture identification 2 panel (BCID2 panel, Biomeriux) tests were also requested on D1. Routine investigation parameter results are shown in Table 1.

**Table 1:** Details of different investigation parameter results ESR: Erythrocyte sedimentation rate, CRP: C-reactive protein, SGOT: Serum glutamicoxaloacetic transaminase, SGPT: Serum glutamic pyruvic transaminase, PT/INR: Prothrombin time/International normalized ratio, APTT: activated partial thromboplastin time, LDH: Lactate dehydrogenase

Parameters	Day 1	Day 2	Day 3	Day4	Day5	Day6	Day7	Day 8
Haemoglobin (12-14 g/dL)	7.8	5.7	6.9	6.6	5.9	9.9	10.7	11.1
RBC count (4-5 x 10^6^/µl)	2.72	1.83	2.34	2.28	2.02	3.38	3.67	3.76
Total WBC count (4-11x10^3^/µl)	11.2	12.8	14.11	8.8	9.1	8.76	11.11	18.4
Differential count	-	N85 L5	N88 L6	N86 L 9	N79 L14	N68 L18	N71 L15	N75 L13
Platelet count (150-400x10^3^/µl)	50	30	50	62	88	90	133	192
Procalcitonin/ Inflammatory.markers	138.4	-	73.25	-	-	5.89	-	ESR 48 CRP 34
SGOT (14-36U/L)	237 U/L	230	126	-	-	45	-	35
SGPT (<35U/L)	70U/L	42	45	-	-	27	-	17
Blood urea (15-36 mg/dL)	30	48	60	56	37	31	27	25
S. creatinine (0.52-1.04 mg/dL)	1.9	2	1.7	1.5	1.4	0.9	0.8	0.8
PT/INR (Control-13.8/INR 0.8-1.2)	>180	21.7/1.76	17.9/1.46	-	14.6/ 1.06	11.5/ 0.95	-	-
APTT (Control 30.0)	142.3	38.3	33.5	-	-	-	-	-
LDH IU/L (120-246 IU/L)	1654	-	-	546	-	-	-	385
S. Calcium (8.8-10.6 mg/dL)	6.8	7.2	7.2	-	-	8.4	-	8.2
Fibrinogen (200-400 mg/dL)	22	127	329	-	567	-	-	-
Protein (6.3-8.2 g/dL)	5	4.6	5.5	-	-	6.3	-	6.9

**Figure 1:**
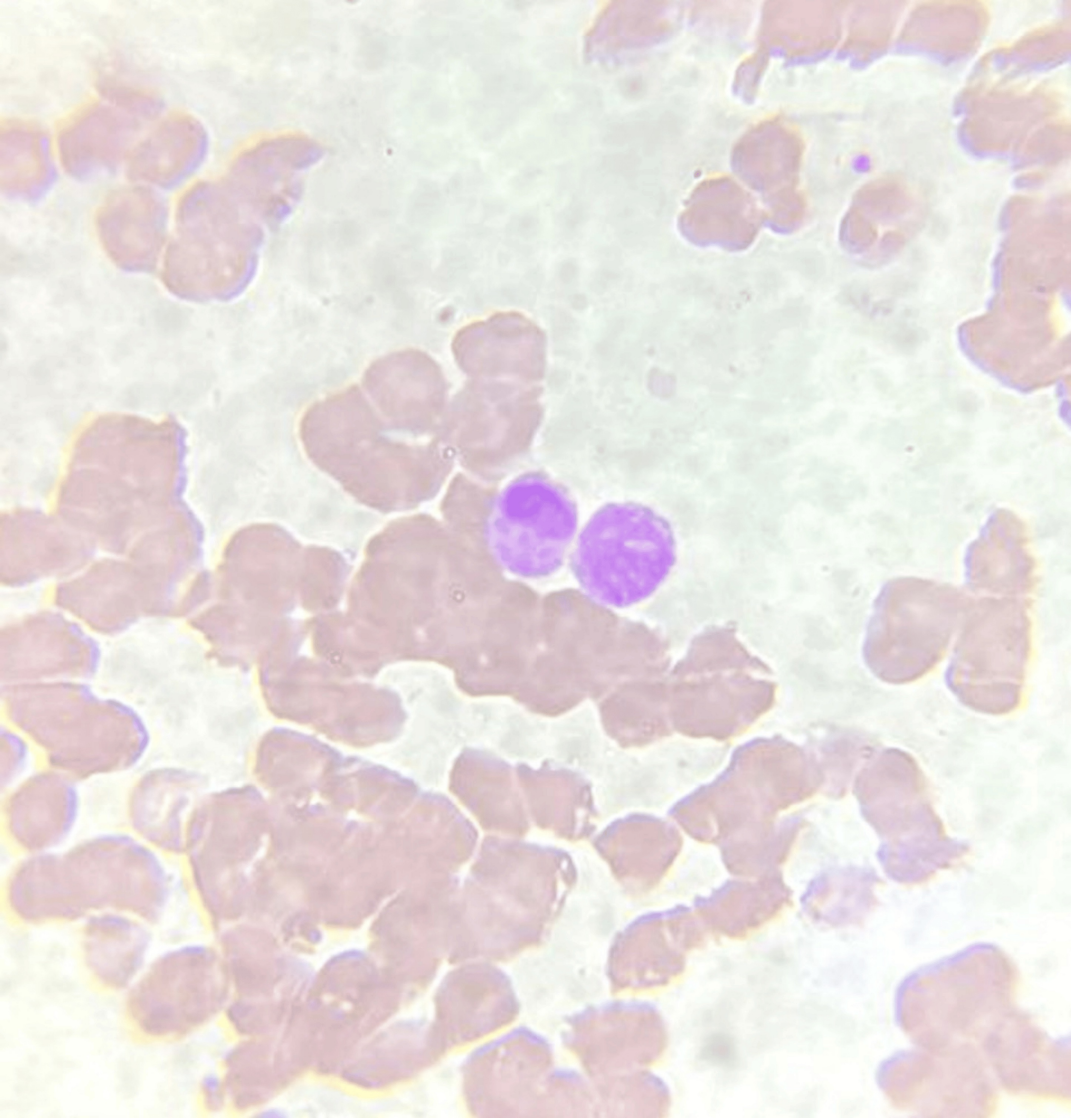
Peripheral blood smear showing leukoerythroblastic picture

Peripheral blood smear (PBS) showed fragmented RBCs with polychromasia suggestive of compensated microangiopathic haemolysis (Figure 1). ADAMTS13 activity test to rule out thrombotic thrombocytopenic purpura was normal. Autoimmune screening yielded negative results. MRI done showed no signs of meningoencephalitis. The disseminated intravascular coagulopathy (DIC) and drop in haemoglobin levels were corrected by multiple units of blood component transfusions, including fresh frozen plasma, packed red cells, and platelet concentrates. 

**Figure 2:**
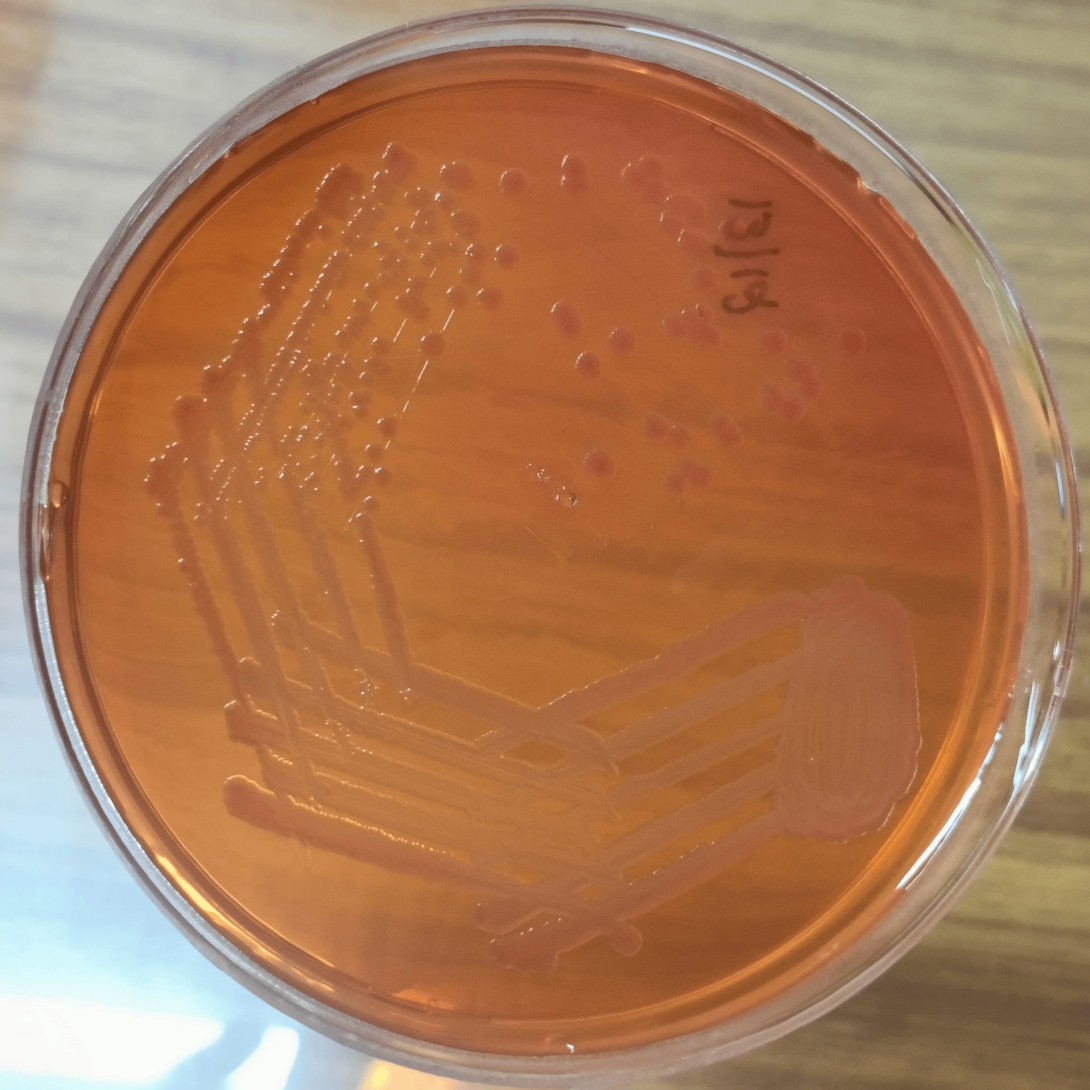
Mac Conkey agar with non-lactose fermenting colonies

The patient had two episodes of loose stools after admission, and the sample was sent for a BioFire film array gastrointestinal panel (GI panel) to exclude toxigenic diarrhea pathogens known to induce coagulopathy. GI panel detected Salmonella species. Paired blood samples flagged positive in BacT/Alert after 15 hours of incubation for Gram-negative bacilli. BioFire film array BCID2 panel report also came as Salmonella species. The routine blood culture yielded *Salmonella Typhi* susceptible to ampicillin, ceftriaxone, cefixime, chloramphenicol, azithromycin, meropenem, trimethoprim/sulfamethoxazole and resistant to ciprofloxacin (Figure 2). The diagnosis of *Salmonella Typhi *sepsis with MODS and DIC directed to start a targeted therapy with ceftriaxone and cotrimoxazole from D2 onwards.

Investigations repeated on D9 were within normal ranges, and the patient had no further episodes of fever spikes. She was discharged on D12 with advice to continue cotrimoxazole till D14. No neurological sequelae were noted during the follow-up visit of the patient after two weeks.

## Discussion

Enteric fever, a potentially fatal multi-systemic febrile disease caused by *Salmonella Typhi*, is transmitted through the faeco-oral route. The bacteria cause various clinical manifestations, including bacteremia, gastroenteritis, fever, and malaise [3, 4, 7].

Sepsis due to *Salmonella Typhi *is uncommon. A case of Salmonella sepsis reported by Adu-Gyamfi R et al. in the year 2019 had a haemodynamically compromised patient presenting with a 10-day history of abdominal pain, vomiting, and diarrhoea. Laboratory investigation showed septicaemia [14]. Another case of typhoid fever reported by Nurnaningsih et al. was complicated by sepsis and DIC. Although DIC scores suggest an imbalance between coagulation and fibrinolysis and are found to be elevated in patients with typhoid fever, they are usually subclinical and rarely account for severe bleeding complications [13]. 

Another sign of severe disease is thrombocytopenia, the mechanism of which is still not fully understood. An estimated 18%-44.9% of patients with typhoid fever suffer from thrombocytopenia and have a higher risk for the development of complications like intestinal perforation, hemolytic uremic syndrome, intracranial hemorrhage, and multi-organ failure [13-15]. 

Our patient manifested with abnormal coagulation, thrombocytopenia, and multiorgan dysfunction. The increased lactate dehydrogenase (LDH), increased reticulocyte percentage, and presence of fragmented RBCs with polychromasia in PBS were suggestive of compensated microangiopathic hemolytic anemia (MAHA), which can be present in both DIC and thrombotic microangiopathy (TMA). In suspicious TMA cases in the context of DIC, further investigations of Shiga-toxins, complements, ADAMTS13 activity, and inhibitors are recommended [16]. In our case, ADAMTS13 activity and microbiological testing (BioFire GI panel) helped us to rule out the causes of TMA like thrombotic thrombocytopenic purpura (TTP), Shiga toxin-producing *Escherichia coli*-associated hemolytic uremic syndrome (STEC-HUS), and atypical HUS (aHUS).

Conventional microbiological diagnostic methods like culture require a long turnaround time and reduce the early execution of specific therapy. Rapid multiplex molecular assays have a rapid turnaround time and high sensitivity, enabling swift identification of infectious agents. In our experience, the detection of *S. Typhi *by means of the BioFire BCID2 panel can rapidly and accurately influence the timely initiation of therapy. Driven by multiplex PCR as a new molecular diagnostic revolution in the management of such complex infections, clinicians are beating the odds of conventional diagnostic methods [17].

The appearance of MDR and XDR strains of *S. Typhi *has complicated the treatment landscape, especially in endemic regions, where resistance to first-line agents such as ampicillin, chloramphenicol, and trimethoprim-sulfamethoxazole is widespread [3, 6, 18]. In severe cases like ours, ceftriaxone remains the drug of choice, although reports of resistance to third-generation cephalosporins are increasing. The merger of cotrimoxazole into the scheme had further synergistic coverage in relation to the global recommendations for controlling severe typhoid fever [19].

Prompt diagnosis, targeted treatment with multidisciplinary management, and supportive measures such as transfusion therapy for DIC contributed significantly to the favourable outcome, although several complications were present in this case. It reinforces the need to familiarize clinicians with the atypical and more severe presentations of typhoid fever, especially in endemic areas.

Preventive measures such as improved sanitation, access to clean water, and vaccination programs still persist in being the cornerstones of the global burden of typhoid fever. Typhoid conjugate vaccines are proving to be a beacon of hope in high-risk populations with long-term immunity and reduction of disease transmission. However, issues like vaccine hesitancy and limited access in low-income settings must be addressed before they will reach global control of typhoid disease [6, 20].

## Conclusions

This case report highlights the importance of prompt identification of severe complications arising in typhoid fever. Life-threatening sepsis and DIC, though rare, require rapid diagnosis and treatment. Modern molecular diagnostics and targeted therapies can significantly improve the prognosis in typhoid fever and its associated complications. But there is still a long path ahead, considering public health interventions and vaccination in order to alleviate this disease in endemic areas as well as at the global level. The multidisciplinary approach we have followed is a stark reminder to improve outcomes for patients, especially in resource-poor settings.
